# Supplementary material for: The ‘Reading the mind in the Eyes' test and emotional intelligence
Source: R Soc Open Sci. 2020 Sep 16;7(9):201305. doi: 10.1098/rsos.201305 (PMC7540806; doi:10.1098/rsos.201305)
Supplement: Appendix [file rsos201305supp1.docx]

Table S1. Results for the moderation analyses including gender as a moderator of the relationship between MSCEIT (total and branches) and Eyes Test performance (total, easy, and difficult items).

| Predictor | Moderator | Criterion | Interaction  coefficient | *SE* | *t* | 95% CI  [lower, upper] |
| --- | --- | --- | --- | --- | --- | --- |
| MSCEIT total | Gender | Eye Test total | 0.0015 | 0.0009 | 1.67 | [-0.0003, 0.0032] |
| MSCEIT total | Gender | Eye Test  easy items | 0.0012 | 0.0010 | 1.18 | [-0.0008, 0.0033] |
| MSCEIT total | Gender | Eye Test difficult items | 0.0017 | 0.0012 | 1.41 | [-0.0007, 0.0041] |
| MSCEIT perceiving | Gender | Eye Test total | 0.0006 | 0.0007 | 0.94 | [-0,0007, 0.0020] |
| MSCEIT perceiving | Gender | Eye Test  easy items | 0.0001 | 0.0008 | 0.12 | [-0.0015, 0.0017] |
| MSCEIT perceiving | Gender | Eye Test difficult items | 0.0012 | 0.0009 | 1.29 | [-0.0006, 0.0030] |
| MSCEIT facilitating | Gender | Eye Test total | 0.0007 | 0.0008 | 0.82 | [-0.0009, 0.0022] |
| MSCEIT facilitating | Gender | Eye Test  easy items | 0.0009 | 0.0010 | 0.97 | [-0.0009, 0.0028] |
| MSCEIT facilitating | Gender | Eye Test difficult items | 0.0004 | 0.0011 | 0.39 | [-0.0017, 0.0026] |
| MSCEIT understanding | Gender | Eye Test total | 0.0015 | 0.0008 | 1.78 | [-0.0001, 0.0031] |
| MSCEIT understanding | Gender | Eye Test  easy items | 0.0009 | 0.0010 | 0.92 | [-0.0010, 0.0029] |
| MSCEIT understanding | Gender | Eye Test difficult items | 0.0020 | 0.0012 | 1.75 | [-0.0002, 0.0043] |
| MSCEIT managing | Gender | Eye Test total | 0.0006 | 0.0006 | 0.91 | [-0.0006, 0.0018] |
| MSCEIT managing | Gender | Eye Test  easy items | 0.0010 | 0.0007 | 1.30 | [-0.0005, 0.0024] |
| MSCEIT managing | Gender | Eye Test difficult items | 0.0002 | 0.0008 | 0.23 | [-0.0014, 0.0018] |

Table S2. Statistical results for the stepwise regression analysis on Eyes Test total.

| Predictors | *R^2^* | *R^2^* adjusted | B | SE | *β* | t | *p* |
| --- | --- | --- | --- | --- | --- | --- | --- |
| Model 1 (Step 1) | .10 | .10 |  |  |  |  |  |
| Understanding |  |  | .004 | .0004 | .32 | 9.86 | < .001 |
| Constant |  |  | .355 | .039 |  | 9.06 | < .001 |
| Model 2 (Step 2) | .11 | .11 |  |  |  |  |  |
| Understanding |  |  | .003 | .0004 | .30 | 9.23 | < .001 |
| Perceiving |  |  | .001 | .0003 | .11 | 3.27 | < .01 |
| Constant |  |  | .280 | .045 |  | 6.18 | < .001 |
| Model 3 (Step 3)* | .12 | .11 |  |  |  |  |  |
| Understanding |  |  | .003 | .0004 | .28 | 8.71 | < .001 |
| Perceiving |  |  | .001 | .0003 | .09 | 2.91 | < .01 |
| Managing |  |  | .001 | .0003 | .08 | 2.50 | .01 |
| Constant |  |  | .236 | .048 |  | 4.88 | < .001 |
| *Best fitting model. | | | | | | | |

Table S3. Statistical results for the stepwise regression analysis on Eyes Test easy items.

| Predictors | *R^2^* | *R^2^* adjusted | B | SE | *β* | t | *p* |
| --- | --- | --- | --- | --- | --- | --- | --- |
| Model 1 (Step 1) | .09 | .09 |  |  |  |  |  |
| Understanding |  |  | .004 | .0004 | .29 | 9.05 | < .001 |
| Constant |  |  | .379 | .047 |  | 7.99 | < .001 |
| Model 2 (Step 2) | .10 | .10 |  |  |  |  |  |
| Understanding |  |  | .004 | .0004 | .27 | 8.34 | < .001 |
| Perceiving |  |  | .001 | .0003 | .13 | 3.92 | < .001 |
| Constant |  |  | .270 | .055 |  | 4.94 | < .001 |
| Model 3 (Step 3)* | .11 | .10 |  |  |  |  |  |
| Understanding |  |  | .003 | .0004 | .26 | 7.88 | < .001 |
| Perceiving |  |  | .001 | .0004 | .12 | 3.60 | < .001 |
| Managing |  |  | .001 | .0003 | .07 | 2.13 | .03 |
| Constant |  |  | .225 | .058 |  | 3.85 | < .001 |
| *Best fitting model. | | | | | | | |

Table S4. Statistical results for the stepwise regression analysis on Eyes Test difficult items.

| Predictors | *R^2^* | *R^2^* adjusted | B | SE | *β* | *t* | *p* |
| --- | --- | --- | --- | --- | --- | --- | --- |
| Model 1 (Step 1)* | .04 | .04 |  |  |  |  |  |
| Understanding |  |  | .003 | .0005 | .21 | 6.31 | < .001 |
| Constant |  |  | .334 | .054 |  | 6.16 | < .001 |
| *Best fitting model. | | | | | | | |
